# Supplementary material for: Lifestyle behaviours and associated factors among people with type 2 diabetes attending a diabetes clinic in Ningbo, China: A cross-sectional study
Source: PLoS One. 2023 Nov 21;18(11):e0294245. doi: 10.1371/journal.pone.0294245 (PMC10662728; doi:10.1371/journal.pone.0294245)
Supplement: S1 Checklist — PLOS ONE Checklist. (DOCX) [file pone.0294245.s001.docx]

Inclusivity in global research

PLOS’ policy on inclusivity in global research aims to improve transparency in the reporting of research performed outside of researchers’ own country or community and ensures that PLOS publications reporting global research adhere to high standards for research ethics and authorship. Authors of relevant research articles may be asked to complete the questionnaire below, which outlines ethical, cultural, and scientific considerations specific to inclusivity in global research. This questionnaire may be requested when researchers have travelled to a different country to conduct research, if research uses samples collected in another country, research with Indigenous populations or their lands, or if research is on cultural artefacts. Researchers travelling to another country solely to use laboratory equipment will not normally be required to complete the questionnaire. However, the questionnaire can be requested at the journal’s discretion for any submission – if you have been requested to complete this questionnaire by the PLOS journal you submitted to, please do so.

Please complete the questionnaire below and include this as a Supporting Information file with your manuscript. Note that if your paper is accepted for publication, this checklist will be published with your article in the supporting information files. Please ensure that you reference the checklist in the main body of your manuscript. We suggest adding a subsection ‘Inclusivity in global research’ to your Methods section and adding the following sentence: “Additional information regarding the ethical, cultural, and scientific considerations specific to inclusivity in global research is included in the Supporting Information (SX Checklist)”

The questions have been designed to be applicable to a wide range of study types, and there are subsections for both human subjects research and non-human subjects research. If any of the questions are not relevant to your research please mark them as “N/A” as appropriate.

**Ethical considerations, permits and authorship**

*This section is applicable to all research types.*

Provide details as to who granted permissions and/or consent for the study to take place in the Methods section of your manuscript. This should include the names of **all** ethics boards, governmental organizations, community leaders or other bodies that provided approval for the study. If individuals provided approval refer to these people by their role or title but do not list their name(s).

Lines 121-126 main manuscript, methods section.

If there were any deviations from the study protocol after approval was obtained please provide details of these changes in the Methods section of your manuscript.

N/A

Did this study involve local collaborators that are residents of the country where the research was conducted or members of the community studied? If you do not have any authors from said communities, please provide an explanation for this below.

Yes, this study involved local collaboration with residents of the country where the research was conducted. Based on previous consultations with key stakeholders, type 2 diabetes was selected as a research priority because of the large burden of type 2 diabetes in Ningbo. The University of Nottingham has campuses both in Nottingham, UK and Ningbo, China. This work was a collaboration between researchers at both sites, and colleagues at the First Affiliated Hospital of Ningbo University. The authorship reflects this, and several of the authors are local clinicians in Ningbo.

Everyone listed as an author should meet PLOS’ criteria for authorship and all individuals who meet these criteria should be included in the author byline, rather than the acknowledgements. Authorship criteria is based on the International Committee of Medical Journal Editors (ICMJE) Uniform Requirements for Manuscripts Submitted to Biomedical Journals - for further information please see here: <https://journals.plos.org/plosone/s/authorship>.

**Human subjects research (e.g. health research, medical research, cross-cultural psychology)**

Did you obtain written informed consent from a representative of the local community or region before the research took place? How did you establish who speaks for the community? Details of written informed consent obtained from study participants should be reported separately in the Methods section of your manuscript.

Ethics approval was received from the Research Ethics Committee of the First Affiliated Hospital of Ningbo University, Ningbo, China (ref. 2019-R057), which is the hospital from which the participant sample was obtained. Details of the process for obtaining written informed consent from participants are provided in 127-131 of the manuscript in the methods section.

How did members of the local community provide input on the aims of the research investigation, its methodology, and its anticipated outcome(s)?

We have taken several measures to ensure alignment with the community's expectations and needs throughout the research process, and several of the authors are local clinicians. The questionnaire was developed and pretested among six local people living with type 2 diabetes from the clinic (who were not included in this study).

Ethical Review Application: We submitted an ethical review application to the ethics committee, including the ethical application form, research proposal, informed consent documents, and resumes of key research personnel. This initial submission outlined our commitment to ethical considerations and adherence to established guidelines.

Engagement with Ethics Committee: At a designated time, we presented our research proposal to the ethics committee. During this presentation, we explained the research's objectives, significance, and detailed implementation methods. We actively sought the committee's opinions and recommendations to further enhance the ethical robustness of our study.

Ethics Committee Feedback: The ethics committee provided feedback on the presented content. We carefully considered their input and made revisions based on their suggestions, ensuring the study's feasibility and appropriateness. This iterative process allowed us to address any potential ethical concerns comprehensively.

Ongoing Reporting and Feedback: Throughout the project's implementation, we maintain regular communication with the ethics committee. We provide periodic updates on research progress and preliminary findings, inviting their valuable feedback. This continuous dialogue helps us remain aligned with ethical standards and community expectations.

Final Report of Preliminary Findings: Once we have obtained preliminary research outcomes, we submit a comprehensive written report to the ethics committee. This report not only summarizes the results but also communicates their implications. By sharing our findings with the committee, we ensure transparency and accountability in our research.

In summary, our research framework places a strong emphasis on ethical considerations and community involvement.

When engaging with the local community, how did you ensure that the informed consent documents and other materials could be understood by local stakeholders?

The participant information sheet and consent form were available in Mandarin. The study objective was explained to all the eligible participants, and written informed consent was obtained from those interested in participating. A quantitative questionnaire in Mandarin was used for data collection. The self-reported nonstandardized questions were developed and pretested in six people with T2DM at the clinic from which the participant sample was drawn (not included in this study).

Will the findings of the research be made available in an understandable format to stakeholders in the community where the study was conducted (e.g. via a presentation, summary report, copies of publications, etc.)? Please provide details of how this will be achieved.

The findings of our research will be made accessible to the stakeholders within the community where the study was conducted; the results of the study will be presented to the Ethics Committee in the form of a final report. A research paper will also be submitted.

**Non-human subjects research using specimens/ animals collected as part of the study, or those housed in archival collections. Examples include archaeology, paleontology, botany and zoology.**

Did the permission you obtained from a local authority to perform the study include an agreement on access to outputs and benefit sharing? This may include procedures to enable fair distribution of the benefits and resources arising from the research performed. Please include any details of Prior Informed Consent and Benefit Sharing Agreements obtained. These may be required by field-specific regulations, for example the Convention on Biological Diversity (CBD) and the associated Nagoya Protocol.

Not applicable

If the material used in your study was imported, please A) provide the year it was imported and B) indicate whether permits were obtained to import/export the materials used, C) provide details of any permits obtained. If this information is not available, please indicate this.

Not applicable

If you used archival specimens, please state how the material used in your study was acquired by the institute it is held in and provide details of any permits obtained for the original excavations/ sample collection. If this information is not available, please indicate this.

Not applicable

How was the potential cultural significance of the materials collected in your study to local communities considered in your research design? Were Indigenous peoples and/or local researchers and institutions involved with archaeological excavations / collection of specimens? If so, please provide a description of their involvement.

Not applicable

If your manuscript includes photographs of human remains please indicate whether authors obtained permission from descendants or affiliated cultural communities to do so.

Not applicable
