# Supplementary material for: Lifestyle behaviours and associated factors among people with type 2 diabetes attending a diabetes clinic in Ningbo, China: A cross-sectional study
Source: PLoS One. 2023 Nov 21;18(11):e0294245. doi: 10.1371/journal.pone.0294245 (PMC10662728; doi:10.1371/journal.pone.0294245)
Supplement: S1 File — Sensitivity analysis using waist-to-height ratio (WHtR) to define abdominal obesity instead of waist circumference thresholds. (DOCX) [file pone.0294245.s002.docx]

**Sensitivity analysis**

**Sensitivity analysis using waist-to-height ratio (WHtR) to define abdominal obesity instead of waist circumference thresholds.**

| **Participant characteristics**  **(Total participants = 806)** | | | **Physically active** | | **Healthy diet** | |
| --- | --- | --- | --- | --- | --- | --- |
|  |  |  | **N (%)** | **Unadjusted OR**  **(95% CI) [p-value]** | **N (%)** | **Unadjusted OR**  **(95% CI) [p-value]** |
| Abdominal obesity (N [%]) | No  Yes  Missing | 177 (22.0)  612 (75.9)  17 (2.1) | 150 (84.7)  505 (82.5)  17 (100.0) | Ref  0.85 (0.53-1.33) [0.487]  N/A | 173 (97.7)  598 (97.7)  17 (100.0) | Ref  0.80 (0.18-2.51) [0.276]  N/A |

| **Participant characteristics**  **(Total participants = 806)** | | | **Smoking** | | **Alcohol drinking** | |
| --- | --- | --- | --- | --- | --- | --- |
|  |  |  | **N (%)** | **Unadjusted OR**  **(95% CI) [p-value]** | **N (%)** | **Unadjusted OR**  **(95% CI) [p-value]** |
| Abdominal obesity (N [%]) | No  Yes  Missing | 177 (22.0)  612 (75.9)  17 (2.1) | 48 (27.1)  124 (20.3)  2 (11.8) | Ref  0.69 (0.47-1.02) [0.058]  N/A | 45 (25.4)  214 (39.4)  6 (35.3) | Ref  **1.59 (1.10-2.34) [0.016]**  N/A |

CI = confidence interval, N = number, N/A = not applicable, OR = odds ratio, Ref = reference category. Significant results (p≤0.05 are highlighted in bold). Unadjusted ORs, 95% CIs and p-values calculated using univariate binomial logistic regression via the glm() function in R.

Adjusted OR (95% CI) [p-value] for the association between abdominal obesity (defined by WHtR≥0.5) and alcohol drinking: **2.09 (1.24-3.57) [0.006].**

Adjusted OR, 95% CIs and p-values calculated using multivariate binomial logistic regression via the glm() function in R, adjusted for age, sex, education level, occupation, residence, marital status, health insurance, duration type 2 diabetes, family history type 2 diabetes, controlled type 2 diabetes, body mass index, and smoking status. Variables selected for multivariable regression analysis due to p≤0.2 in univariate analysis.
