# Supplementary material for: Lifestyle behaviours and associated factors among people with type 2 diabetes attending a diabetes clinic in Ningbo, China: A cross-sectional study
Source: PLoS One. 2023 Nov 21;18(11):e0294245. doi: 10.1371/journal.pone.0294245 (PMC10662728; doi:10.1371/journal.pone.0294245)
Supplement: Attachment — Submitted filename: Response to Reviewers.docx [file pone.0294245.s004.docx]

Thursday, 10 August 2023

Dear Hidetaka Hamasaki

**Re: PONE-D-23-19169**

**Lifestyle behaviours and associated factors among people with type 2 diabetes in Ningbo, China: A cross-sectional study**

I write on behalf of my co-authors to thank the editor and reviewers for their careful review, and appreciate the opportunity to respond with a revised submission.

Please see point-by-point responses below. Please note line numbers refer to the tracked-changes version of the manuscript.

Yours Sincerely,

Dr Kaushik Chattopadhyay, on behalf of the authors

**Editor**

**Comment #1:** Please ensure that your manuscript meets PLOS ONE's style requirements, including those for file naming

**Author’s Response:** PLOS ONE’s style requirements followed and files renamed as per the guidance.

**Comment #2:** In the ethics statement in the Methods, you have specified that verbal consent was obtained. Please provide additional details regarding how this consent was documented and witnessed, and state whether this was approved by the IRB

**Author’s Response:** Apologies for any confusion - written informed consent was obtained from participants, not verbal. We cannot find any references to verbal consent in the manuscript. References to written consent are available in lines 79-80 and 133 of the manuscript (unchanged).

**Comment #3:** Please include a complete copy of PLOS' questionnaire on inclusivity in global research in your revised manuscript.

**Author’s Response:** This has been completed and enclosed as supplementary file 1: S1 Checklist. It is also now referenced in the manuscript as below:

Lines 135-137: Additional information regarding the ethical, cultural, and scientific considerations specific to inclusivity in global research is included in the Supporting Information (Appendix 1: S1 Checklist)

**Comment #4:** the editor thinks that the authors should describe the sampling method used in this study and discuss potential biases in the manuscript.

**Author’s Response:** Information on the sampling strategy has been added to the methods section (edited text is provided below – eligible clinic patients were approached consecutively by the physicians during the study period and invited to participate). The discussion has been expanded to highlight potential selection biases present in the study, and how they might have influenced the findings, as well as issues of sample representativeness and generalisability. The track-changes demonstrating these changes are presented below. Social desirability bias has also been discussed in lines 304-307.

Lines 73-77: **Sampling strategy**. At least 784 participants were needed based on a 95% confidence level, a 3.5% margin of error, and a conservative population proportion of 50% (17). During the time period of the study, eligible clinic patients were approached consecutively by the physicians and invited to participate. No incentives to participate (financial or otherwise) were provided.

Lines 316-326: Given the highly contextual nature of lifestyle behaviours, the generalisability of the findings outside of the Ningbo context or to those without T2DM is limited. Further, the clinic-based sampling strategy created potential selection bias, as the characteristics of participants attending the diabetes clinic may differ systematically from people living with T2DM more generally across Ningbo. Additionally, those from the clinic who opted to participate may differ from those who did not, and data on non-responders were not collected due to the large workload in the busy clinic setting of the study. It is possible that people living with T2DM who were engaged and satisfied with clinic care and willing to take part in a study may be more likely to also engage with healthy lifestyle advice, which could have resulted in an overestimate of healthy lifestyle behaviours in our sample.

Lines 333-334: This study has demonstrated the prevalence of four key lifestyle behaviours amongst people living with T2DM attending a diabetes clinic in Ningbo, China.

**Reviewer 1**

**Comment #1:** Line 66-68: The study participants were selected from single hospital, which indicated that the study participants can not represent the status of the total T2DM patients living in Ningbo. In other words, I considered that there may be serious and uncontrolled representativeness bias in the present study.

**Author’s Response:** This is an important point and has been addressed in the reply to Reviewer 1 comment #8 below.

**Comment #2:** Line 74: The diagnostic criteria of T2DM should be described in the text. Is there difference between diagnostic criteria of T2DM of China and of IDF / ADA? If there is, then why?

**Author’s Response:** Apologies, on review of the manuscript there was an error in the reference order so that reference 18 did not correspond to the Chinese type 2 diabetes guideline. This has now been corrected. The Chinese guideline in use at the time corresponded with standard WHO guidance on diagnosing type 2 diabetes, which has been added for clarity. We have not added the full diagnostic criteria given these are referenced and widely accepted, however we would be happy to add these if required by the editor and reviewers.

Lines 80-81: The diagnosis of T2DM was based on the Chinese guideline, which was in keeping with World Health Organization recommendations for diagnosis (18).

18.	Jia W, Weng J, Zhu D, Ji L, Lu J, Zhou Z, et al. Chinese Diabetes Society. Standards of medical care for type 2 diabetes in China. Diabetes Metab Res Rev. 2019; 35:e3158.

**Comment #3:** Line 88-89: The authors should clearly describe if there were study participants whose two readings were significantly different, and the significant difference should also be defined. If there were, then the measurement may be untrusted and that patient should be excluded.

**Author’s Response:** During data cleaning, there was no pre-set threshold for a significant difference between two measurements, however data were screened for implausible values, and these were deleted where found and treated as missing. This was done for: HbA1C (3 participants), duration type 2 diabetes (1 participant), UKDDQ Q16 (1 participant), UKDDQ Q17 (1 participant), UKDDQ Q18 (1 participant), UKDDQ Q19 (1 participant), and IPAQ Q1 (4 participants). If the implausible value was one of a pair of repeated measures, both measures were deleted and treated as missing. This was done for: systolic blood pressure (1 participant), body weight (2 participants), and height (1 participant).

Lines 140-142: During data cleaning, data were screened for implausible values, and these were deleted where found and treated as missing. If the implausible value was one of a pair of repeated measures, both measures were deleted and treated as missing.

**Comment #4:** Line 101-103, table 1 and 2: I suggest that waist-to-height ratio (WHtR) should be used to define abdominal obesity because waist circumference alone did not take differences in height into account. At least, the associations of WHtR with lifestyle behaviours and associated factors should be researched in the present manuscript.

**Author’s Response:** Thank you for this suggestion. In response, we have conducted a sensitivity analysis using waist-to-height ratio (WHtR) to define abdominal obesity instead of waist circumference thresholds and the results of this have been added to the submission as appendix 2 (S2 File), and discussed in the manuscript as follows:

Lines 155-156: A sensitivity analysis was conducted using waist-to-height ratio (WHtR) ≥ 0.5 to define abdominal obesity rather than the previously specified waist circumference thresholds (23).

Lines 196-199: In sensitivity analysis using WHtR ≥ 0.5 to define abdominal obesity (rather than the previously specified waist circumference thresholds) being an alcohol drinker was significantly associated with abdominal obesity in the adjusted analysis (OR 2.09, 95% CI 1.24-3.57) [Appendix 2: S2 File].

Lines 291-297: In sensitivity analysis, using WHtR ≥ 0.5 to define abdominal obesity (rather than the previously specified waist circumference thresholds), being an alcohol drinker was significantly associated with abdominal obesity (adjusted OR 2.09, 95% CI 1.24-3.57). This may be a more sensitive measure of abdominal obesity compared to waist circumference thresholds which do not take height into account, and is an intuitive finding given the calorific content of alcohol (40).

Please also see Appendix 2 - S2 File

**Comment #5:** Line 136: The study should include information on duration of smoking cessation in those ex-smokers. Actually, the years immediately following smoking cessation may represent a time of increased risk for DM (PMID: 34964876).

**Author’s Response:** Thank you for this suggestion, and we acknowledge the importance of ex-smoker status as a risk factor for a wide variety of poor health outcomes. Data were collected on whether participants had ever smoked and duration of smoking cessation if they had quit, however the primary aim of this manuscript was to describe the prevalence of current healthy and unhealthy lifestyle behaviours and the factors associated with them. We opted to look at point prevalence of these lifestyle factors, focusing on those participants who were current smokers, current alcohol drinkers, currently physically active or inactive, and who currently had healthy or unhealthy diets. The rationale for this is that past unhealthy lifestyle behaviours are not modifiable, whereas current unhealthy lifestyle behaviours potentially are. We have added additional information to the methods to make this rationale clear, as described below.

Lines 115-118: This paper focused on current rather than retrospective healthy and unhealthy lifestyle behaviours based on the rationale that these are potentially modifiable, whereas past lifestyle behaviours are not.

**Comment #6:** Line 207-208: It is difficult to exactly assess the intensity of physical activity (PA). The present study obtained PA data using IPAQ questionnaire (Line 109). I guess there may be another reasons: for example, the authors should describe if there was difference in education level between rural and urban participants in the results section. If education level of rural participants was lower than their urban counterparts, than the reliability of their IPAQ questionnaire results may be lower because of lower ability to understand and/or patience.

**Author’s Response:** Many thanks, this has been incorporated into the discussion as follows:

Lines 244-249: An alternative explanation may be differential validity of the IPAQ questionnaire between the urban and rural participants of the study. For example, 25.3% of participants from urban areas had college or university education, versus 6.2% in rural areas. Participants with fewer educational opportunities could have reduced understanding of the required questionnaire responses.

**Comment #7:** Line 233-241: The association between alcohol drinking and DM should be discussed in more detail. Moderate alcohol intake had minimal detrimental effects on long-term blood glucose management (PMID: 26294775). Furthermore, though the authors of a recent published study (PMID: 37414658) speculated that the emerged discomfort reduced alcohol consumption frequency in DM population of China, that frequency was not significantly associated with DM risk.

**Author’s Response:** The two suggested references seem to outline risk factors for diabetes development rather than diabetes control, which is not the focus of this paper which looks at lifestyle of people with existing T2DM. However, the two suggested references have been incorporated into the discussion as suggested to try and example some of nuance regarding the relationship between alcohol and diabetes. Please see the relevant edit below.

Lines 264-266: Evidence for whether alcohol is an independent risk factor for T2DM is mixed and may interact with other factors such as gender and ethnicity (36,37). However, alcohol consumption can increase the risk of hypoglycaemia and can negatively impact self-care behaviours (13,14).

36.	Knott C, Bell S, Britton A. Alcohol Consumption and the Risk of Type 2 Diabetes: A Systematic Review and Dose-Response Meta-analysis of More Than 1.9 Million Individuals From 38 Observational Studies. Diabetes Care. 2015;38(9):1804-12.

37.	Gao YX, Man Q, Zhang J. The association of prediabetes with dietary patterns, life behavior and cardiovascular risk factors among adult population without previously diagnosed non-communicable diseases. Nutr Metab Cardiovasc Dis. 2023:S0939-4753(23)00091-1.

**Comment #8: Line 264-265:** Though that may be the first study "in a cohort of patients living with T2DM in Ningbo", I still considered that the innovativeness of the present study is quite limited. As the authors mentioned, Ningbo ist an economically developed city in the eastern, coastal province of Chekiang (Line 47), however, I don't understand why the authors selected Ningbo as the studied city. The status of the city can not represent the most developed regions of China, and that city may not even represent the province of Chekiang. Furthermore, as above-mentioned, the study participants of the present study can not represent the status of the total T2DM patients living in Ningbo. Therefore, I considered that the present study may be only beneficial to those 806 study participants, and other T2DM patients may not benefit from the present study. The authors should clearly explain the innovativeness of the present study and the representativeness of the sample.

**Author’s Response:** The study site was chosen based on an existing collaborative partnership between the University of Nottingham campus in Ningbo, China, and the First Affiliated Hospital of Ningbo University, Ningbo, China. Based on previous consultations with key stakeholders, type 2 diabetes was selected as a research priority because of the large burden of type 2 diabetes in Ningbo. The study was funded by the Nottingham China Health Institute, China, and the Major Science and Technology Projects for Health of Zhejiang Province (Grant No. WKJ-ZJ-2216). Rationale for study site selection has been added to the methods for clarity (please see edits below).

The point about sample representativeness and generalisability is important, as the characteristics of participants attending the diabetes clinic in this study may differ from people living with T2DM more generally across Ningbo. We have edited the manuscript to caveat generalisability more carefully. In addition, the phrase ‘people living with T2DM in Ningbo, China’ has been changed throughout to ‘people living with T2DM attending a diabetes clinic in Ningbo, China’. However, we would argue the findings of the study are still worth reporting and sharing, and the learning from the present study can be used to inform future lifestyle behaviour support in this specific clinic context, aided by the aforementioned collaborations. As well as prevalence estimates for the lifestyle behaviours the study also provides important information on the factors associated with these lifestyle behaviours.

Lines 68-72: The study site was chosen based on an existing collaborative partnership between the University of Nottingham campus in Ningbo, China, and the First Affiliated Hospital of Ningbo University, Ningbo, China; which is a tertiary care hospital including a diabetes centre with a team of qualified and experienced diabetes experts (16).

Lines 21-24: In order to describe the prevalence of being physically active, having a healthy diet, current smoking, and current alcohol drinking among people living with T2DM attending a diabetes clinic in Ningbo, China, a cross-sectional survey was undertaken.

Lines 34-35: Specific interventions targeting smoking cessation and reduction in alcohol drinking may be of benefit to men living with T2DM attending a diabetes clinic in Ningbo.

Lines 59-62: Therefore, we aimed to describe the prevalence of being physically active, having a healthy diet, current smoking, and current alcohol drinking amongst people living with T2DM attending a diabetes clinic in Ningbo, China; and to determine the factors associated with these lifestyle behaviours.

Lines 298-301: To the authors’ knowledge, this is the first study addressing the prevalence of lifestyle behaviours in a cohort of patients living with T2DM at a diabetes clinic in Ningbo, China; and may help to inform development of future lifestyle behaviour support interventions at the clinic.

Lines 318-326: Given the highly contextual nature of lifestyle behaviours, the generalisability of the findings outside of the Ningbo context or to those without T2DM is limited. Further, the clinic-based sampling strategy created potential selection bias, as the characteristics of participants attending the diabetes clinic may differ systematically from people living with T2DM more generally across Ningbo. Additionally, those from the clinic who opted to participate may differ from those who did not, and data on non-responders were not collected due to the large workload in the busy clinic setting of the study. It could be anticipated that people living with T2DM who were engaged and satisfied with clinic care and willing to take part in a study may be more likely to also engage with healthy lifestyle advice, which could have resulted in an overestimate of healthy lifestyle behaviours in our sample. However, the study provides useful information in a specified population helping to identify potential areas for targeted lifestyle interventions.

Lines 333-334: This study has demonstrated the prevalence of four key lifestyle behaviours amongst people living with T2DM attending a diabetes clinic in Ningbo, China.

Lines 341-343: Specific interventions targeting smoking cessation and reduction in alcohol drinking may be of benefit to men living with T2DM attending a diabetes clinic in Ningbo.

**Comment #9:** Line 269-281: There were some other limitations that the authors should mention in the text. For example, the cause and effect relationship can not be examined in the present cross-sectional study.

**Author’s Response:** Additional limitations added as per the tracked-changes below:

Lines 308-330: The binary categorisation of the four lifestyle behaviours in the present study comes with some loss of information on extent of lifestyle behaviours, however, was necessary for the chosen analytical strategy. Data were not collected on comorbidities and complications which may also associate with lifestyle behaviours. The cross-sectional nature of the study also prevented establishment of cause and effect between the associated factors and the lifestyle behaviours. In addition, there were a high number of analyses in the present study which may increase the risk of false-positive associations. Those associations of borderline statistical significance should be interpreted with caution. Given the highly contextual nature of lifestyle behaviours, the generalisability of the findings outside of the Ningbo context or to those without T2DM is limited. Further, the clinic-based sampling strategy created potential selection bias, as the characteristics of participants attending the diabetes clinic may differ systematically from people living with T2DM more generally across Ningbo. Additionally, those from the clinic who opted to participate may differ from those who did not, and data on non-responders were not collected due to the large workload in the busy clinic setting of the study. It could be anticipated that people living with T2DM who were engaged and satisfied with clinic care and willing to take part in a study may be more likely to also engage with healthy lifestyle advice, which could have resulted in an overestimate of healthy lifestyle behaviours in our sample. However, the study provides useful information in a specified population helping to identify potential areas for targeted lifestyle interventions. Complementary qualitative research which explores the lived experiences of those with T2DM regarding their lifestyle behaviours would help elucidate barriers and facilitators of lifestyle change in this context.

**Comment #10:** Line 276-278: I still considered that the present study only provides information in those 806 study participants because they can not represent the status of the total T2DM patients living in Ningbo.

**Author’s Response:** Please see response including track-changes for Reviewer 1 – comment #8

**Comment #11:** Line 284-285: Again, the prevalence found in the present study was not the prevalence amongst T2DM patients in Ningbo, but just the prevalence amongst those 806 study participants.

**Author’s Response:** Please see response including track-changes for Reviewer 1 – comment #8

**Reviewer 2**

**Comment #1:** How did you address selection bias?

**Author’s Response:** Eligible clinic patients were approached consecutively by the physicians and invited to participate. No incentives were provided for participation. However, we acknowledge that the study is at risk of selection bias, and this has now been highlighted in the discussion (please see edits below). We have also tried to clarify throughout the manuscript that the prevalence estimates for lifestyle behaviours given in this paper are specific to the clinic population that was studied rather than generalisable to people with type 2 diabetes across Ningbo. The phrase ‘people living with T2DM in Ningbo, China’ has been changed throughout to ‘people living with T2DM attending a diabetes clinic in Ningbo, China’.

Lines 318-326: Further, the clinic-based sampling strategy created potential selection bias, as the characteristics of participants attending the diabetes clinic may differ systematically from people living with T2DM more generally across Ningbo. Additionally, those from the clinic who opted to participate may differ from those who did not, and data on non-responders were not collected due to the large workload in the busy clinic setting of the study. It could be anticipated that people living with T2DM who were engaged and satisfied with clinic care and willing to take part in a study may be more likely to also engage with healthy lifestyle advice, which could have resulted in an overestimate of healthy lifestyle behaviours in our sample

**Comment #2:** How were the participants asked to join the study? Were there any incentives?

**Author’s Response:** During the time period of the study, eligible clinic patients were approached consecutively by the physicians at the clinic and invited to participate. No incentives to participate (financial or otherwise) were provided. This has now been added to the manuscript for clarity (please see below).

Lines 75-77: During the time period of the study, eligible clinic patients were approached consecutively by the physicians and invited to participate. No incentives to participate (financial or otherwise) were provided.

**Comment #3:** How was the response rate? It could very well be that those who accepted to participate are the ones who are more aware of their health. This is especially relevant because anthropometric measurements were taken, which would be uncomfortable for overweight or obese individuals.

**Author’s Response:** Unfortunately, due to the busy workload of staff in the clinic we did not collect data on the number of participants who were approached to take part and declined to participate. This has been added as a limitation of the study in the discussion section (please see edits below).

Lines 318-326: Further, the clinic-based sampling strategy created potential selection bias, as the characteristics of participants attending the diabetes clinic may differ systematically from people living with T2DM more generally across Ningbo. Additionally, those from the clinic who opted to participate may differ from those who did not, and data on non-responders were not collected due to the large workload in the busy clinic setting of the study. It could be anticipated that people living with T2DM who were engaged and satisfied with clinic care and willing to take part in a study may be more likely to also engage with healthy lifestyle advice, which could have resulted in an overestimate of healthy lifestyle behaviours in our sample.

**Comment #4:** Is it possible to perform a non-responder analysis? This might also help understand the results of the study

**Author’s Response:** Please see response to reviewer 2, comment #3 - unfortunately these data were not collected due to the large workload in the busy clinic setting of the study.

**Comment #5:** Did the questionnaire also include question on complications and comorbidities? If no, why, and if yes, why were they not considered in the analysis as they are highly relevant (for example by stratifying the sample by morbidity level)?

**Author’s Response:** Apologies, these data were not collected and this has been added as a study limitation.

Lines 311-312: Data were not collected on comorbidities and complications which may also associate with lifestyle behaviours.

**Comment #6:** Why were the extent of smoking and alcohol (for example number of cigarettes per day or frequency of alcohol intake) not considered?

**Author’s Response:** Data on the extent of smoking and alcohol including number of cigarettes smoked per day and frequency of alcohol intake were collected but were not presented in the current manuscript. We accept that the binary categorisations of the four lifestyle behaviours comes with some loss of information, and have added this as a further study limitation. Whilst it would be interesting to try and assess for a dose-response relationship between some of the associated factors and the lifestyle behaviours, e.g. through categorising people into heavy and light smokers, we opted not to undertake this given the already high number of analyses present and the increased risk of spurious results if multiple additional analyses are added.

Lines 308-311: The binary categorisation of the four lifestyle behaviours in the present study comes with some loss of information on extent of lifestyle behaviours, however was necessary for the chosen analytical strategy.
